# Family support and motivational factors in weight loss among children and adolescents—a cross-sectional study

**DOI:** 10.3389/fnut.2025.1618016

**Published:** 2025-10-27

**Authors:** Małgorzata Wąsacz, Danuta Ochojska, Izabela Sarzyńska, Oliwia Bartkowska, Szymon Stańczyk, Joanna Błajda, Damian Frej, Marta Kopańska

**Affiliations:** ^1^Department of Medical Psychology, Faculty of Medicine, Collegium Medicum, University of Rzeszów, Rzeszów, Poland; ^2^Faculty of Health Sciences and Psychology, Medical College, University of Rzeszów, Rzeszów, Poland; ^3^Student Research Club “Reh-Tech”, Medical College of Rzeszow University, Rzeszów, Poland; ^4^Department of Automotive Engineering and Transport, Kielce University of Technology, Kielce, Poland

**Keywords:** childhood obesity, overweight, motivation, family support, self-esteem, healthy eating habits, emotions, health interventions

## Abstract

**Introduction:**

Childhood and adolescent obesity is an increasing public health concern. The effectiveness of overweight treatment in this population depends on multiple factors, including the level of motivation, emotions associated with behavioral change, and family support.

**Aim:**

The aim of this study was to analyze the levels of motivation, emotions, and self-esteem among overweight and obese children and adolescents, and to identify the potential role of family support in the process of changing dietary habits.

**Materials and methods:**

The study involved 100 participants aged 10 to 18, recruited from three settings: school, hospital, and rehabilitation health resort environments (sanatoria). Inclusion criteria comprised overweight or obesity (BMI ≥ 85th percentile), regular school attendance, and parental consent. Exclusion criteria included chronic illness, metabolic or genetic disorders, and pharmacotherapy affecting body weight. A custom-designed questionnaire was used, along with elements of individual interviews. We hypothesized that higher levels of family support would be associated with greater motivation and longer maintenance of healthy lifestyle changes. The analysis focused on the duration of maintaining healthy habits, eating control, self-esteem, and emotions accompanying weight loss. Statistical analysis included significance tests and group comparisons.

**Results:**

Most participants reported short-term attempts to change habits (1–5 days), and only 16% maintained a diet for 28 days. A significant portion experienced negative emotions such as anxiety, shame, or guilt, particularly among high school students. Additionally, 67% of respondents stated that their body weight influenced their self-perception. A statistically significant relationship was found between BMI category and dissatisfaction with one’s weight (*p* = 0.0261). The interviews revealed the critical role of parental attitudes in sustaining the child’s motivation.

**Conclusion:**

The findings highlight the need to include emotional and family-related components in overweight and obesity treatment programs for children. A comprehensive, individualized approach increases the likelihood of lasting lifestyle changes and improved psychological well-being in young patients.

## 1 Introduction

Childhood overweight and obesity are currently one of the most serious public health threats in the world, both in highly developed and developing countries. According to the World Health Organisation (WHO), since the 1970s, the rate of obesity among children and young people has increased more than tenfold – from 11 million in 1975 to 124 million in 2016, with an additional 213 million children being overweight (1). It is predicted that by 2050, 15.6% (186 million) of children and adolescents aged 5 to 14 and 14.2% (175 million) of people aged 15 to 24 worldwide will struggle with excess body weight, especially obesity (2). This problem also affects Poland - according to national and European studies, almost 30% of school-age children in our country are overweight or obese (3). These include increased likelihood of developing metabolic syndrome, type 2 diabetes, hypertension and cardiovascular complications, as well as psychosocial consequences such as low self-esteem, depressive symptoms and eating disorders, which may impair social functioning (1, 2).

Childhood obesity is not only an aesthetic or social problem, but above all a significant health risk that can lead to serious and long-term consequences. The most common health effects include type 2 diabetes, hypertension, dyslipidaemia, insulin resistance, cardiovascular disease, obstructive sleep apnoea, and liver and osteoarticular diseases (4–7). What is important is that many of these conditions, which until recently were only seen in adults, are now being diagnosed in children and adolescents. Studies show that overweight children show signs of hypertension, fatty liver and psychosocial complications (8).

In addition to the physical aspects, obesity has a huge impact on the mental and social health of a child. Low self-esteem, stigmatisation, depression, anxiety and social exclusion are just some of the emotional problems that overweight children have to face (9, 10). These phenomena have a negative impact on the socialization and education process, can lead to self-destructive behavior and even suicidal thoughts (11, 12). Childhood obesity also correlates with lower educational and professional achievements in adulthood, as well as with a lower quality of life.

The scientific literature clearly indicates that the causes of childhood obesity are multifactorial and complex. Genetic, environmental, psychological, cultural and behavioral factors are among the main determinants. The lifestyle of the whole family also plays an important role - diet, level of physical activity, family relationships and even sleep patterns (13–15). Risk factors already appear in the prenatal period – these include gestational diabetes, maternal obesity before pregnancy, caesarean section, as well as the use of antibiotics during pregnancy (16). In infancy and preschool age, the impact is influenced by the method of feeding (breastfeeding vs. formula feeding), the quality of the diet, sleep and the rate of weight gain (17).

In the later stages of a child’s life, the development of overweight and obesity is influenced by, among other things, poor eating habits (skipping breakfast, snacking, high consumption of sugar and saturated fats), low physical activity, a sedentary lifestyle and spending long periods of time in front of a screen (18, 19). Peer pressure, food advertising and social media content have also been shown to influence the health choices of children and adolescents (20). The family, school, local environment and health policies form a system of influences that can both support and sabotage efforts to prevent obesity (21).

The problem of obesity does not only affect the national population, but also requires regional analyses. One example is the Podkarpackie Voivodeship, which, despite a relatively low level of urbanization, has alarming rates of overweight and obesity in children. According to reports from the Supreme Audit Office, the problem affects more than 22% of children aged 2–18 (22). In the context of therapeutic and preventive measures, increasing importance is being attached to the issue of motivation. Weight loss, especially in childhood, cannot be achieved solely through restrictive diets or externally imposed physical activity. Effective and lasting changes require the child’s emotional involvement, their internal motivation and support from adults - parents, teachers, doctors, psychologists (23, 24). Literature indicates that the higher the level of internal motivation, the greater the chances of success in the weight loss process and maintaining a new lifestyle (25).

Motivation in children is not a homogenous phenomenon – it can be cognitive, emotional or social. Effective motivational strategies include goal-setting techniques, reward systems, adult modelling, group support, multimedia interventions and health apps (26). Social campaigns, school programs and the availability of infrastructure that promotes physical activity (e.g., sports fields, cycle paths, healthy canteens) are also important (27).

Research into the effectiveness of interventions to date shows that the best results are achieved by comprehensive programs lasting at least 6 months, based on cooperation between the family, school and healthcare system. Unfortunately, Poland lacks a nationwide, coherent strategy to combat childhood obesity. Local programs, such as the project for the prevention and early detection of overweight among third-grade primary school pupils, which has been implemented in Rzeszów since 2024, have great potential, but their effectiveness will depend on many factors, including access to specialists, continuity of activities and the level of family involvement.

This study focuses on the analysis of effective methods of shaping the motivation to lose weight among children and adolescents from the Podkarpackie Voivodeship. The aim of the study was to identify the most important factors influencing motivation, to assess the effectiveness of various strategies and to develop practical recommendations for prevention and therapeutic programs. The research was interdisciplinary and included the analysis of medical, psychological, educational and social aspects.

The problem of childhood obesity requires a comprehensive approach that takes into account dietary and physical activity interventions as well as psychological and environmental factors. Strengthening the child’s motivation, sense of agency, and engagement in the process of change is of particular importance. Systemic support from the family, school, and professionals can contribute to more effective prevention and treatment of overweight and obesity among children and adolescents. The present study was designed to investigate motivational and emotional factors connected with weight reduction among overweight and obese children and adolescents, with particular emphasis on the role of family support in this process. The research combined quantitative and qualitative approaches: the quantitative part examined the relationship between BMI, motivation, self-esteem and emotions, while the qualitative part explored the experiences of children and adolescents regarding parental involvement, emotions and barriers to maintaining lifestyle changes. It was assumed that a higher level of family support would be related to greater motivation, longer maintenance of healthy habits and higher self-esteem in overweight and obese children and adolescents.

The aim of the study was to identify motivational factors conducive to weight reduction in 100 children and adolescents with overweight or obesity. The article is structured as follows. The Materials and Methods section describes the study design, research settings, participants, applied tools and statistical procedures. The Results section presents the main findings concerning motivational factors, emotions, self-esteem and the role of family support. In the Discussion, these findings are interpreted in the context of previous literature, with particular attention to the practical implications for prevention and treatment of childhood obesity. The article ends with Conclusions, which summarize the most important results, indicate limitations of the study and outline directions for future research.

The theoretical framework of this study was grounded in Self-Determination Theory (SDT), which differentiates between autonomous and controlled motivation. Autonomous motivation arises from internalized values, personal growth, and perceived competence, whereas controlled motivation is driven by external pressures, expectations, or the desire for approval. According to SDT, behaviors regulated autonomously are more sustainable and adaptive, as they stem from internal commitment rather than external reinforcement. In addition, elements of Social Cognitive Theory (SCT) were considered, emphasizing the role of modeling, self-efficacy, and social support in behavior change. The integration of these perspectives provides a comprehensive understanding of how motivational regulation and family support interact in shaping health-related behaviors among children and adolescents.

## 2 Materials and methods

The study was designed as a cross-sectional, observational and quantitative-qualitative study. The main objective was to determine the factors influencing the motivation to lose weight in children and adolescents and to assess the effectiveness of selected motivational strategies. Due to the nature of the problem and the need to reach a large group of participants, the method of field research carried out in educational and medical institutions in the Podkarpackie Voivodeship was chosen. The choice of the Podkarpackie Voivodeship was dictated by both the availability of the studied population and the reported high rates of overweight and obesity among children in this region (1). The field research was conducted in primary and secondary schools, pediatric clinics and community centers in Rzeszów and neighboring districts.

The research project took into account the diversity of environments - participants were recruited from both urban and rural areas, which allowed for a comparison of the impact of socio-economic factors on motivation and health behaviors.

### 2.1 Ethical aspects

The study was conducted in accordance with all ethical and formal standards. Before the start of the study, the project received a positive opinion from the Bioethics Committee at the Medical University of Rzeszów (approval number: KBET/219/2023). All research procedures were in accordance with the principles of the Helsinki Declaration. Consent to participate in the study was a two-step process: (1) written consent from a parent or legal guardian, and (2) verbal and/or written consent from the child. Participants were informed about the purpose of the study, its course, the possibility of withdrawing at any time, and the guarantee of anonymity. Data was collected and stored in accordance with GDPR regulations, and the results were analyzed in aggregate form.

### 2.2 Participants

The study was cross-sectional, observational and quantitative-qualitative. It was carried out in 2023–2024 in the Podkarpackie Voivodeship, in south-eastern Poland. The aim of the study was to identify motivational factors conducive to weight reduction among overweight or obese children and adolescents. The participants were recruited from three different environments – educational, clinical and therapeutic – which allowed for a multifaceted analysis of health behaviors and motivation in different socio-health contexts. Participants were recruited and assessed in three different institutional settings, which also served as the research sites: (1) the Secondary School in Tyczyn (educational environment), (2) the 2nd Paediatrics, Endocrinology and Paediatric Diabetology Clinic of the Clinical Regional Hospital No. 2 in Rzeszów (clinical environment), and (3) the Zimowit Health Resort in Rymanów-Zdrój (therapeutic/rehabilitation environment). No participants with chronic illnesses, eating disorders, or pharmacological treatment affecting weight were included.

In each of these facilities, anthropometric measurements and questionnaire surveys were conducted on site, using the same portable, standardized equipment and following identical procedures. This ensured methodological consistency and allowed for valid comparison across settings, while still capturing the diversity of socio-health environments in which children and adolescents function.

Anthropometric measurements were conducted by trained medical staff using portable, standardized equipment (calibrated scales and stadiometers), and the data collection took place in classrooms specially prepared for this purpose. Questionnaires were administered in the same setting immediately after the measurements, under the supervision of the research team, which guaranteed comparable conditions for all participants regardless of recruitment source. This procedure ensured methodological consistency and addressed potential concerns about differences between research sites.

Children and adolescents aged 10 to 18 years were qualified for the study, who met the inclusion criteria:

1. BMI index ≥ 85th percentile for age and gender according to WHO standards.
2. No diagnosed metabolic diseases (e.g., type 1 diabetes) or genetic obesity syndromes (e.g., Prader-Willi syndrome).
3. No use of pharmacotherapy affecting body weight.
4. Informed consent by parents/legal guardians and by the child.

Although inclusion targeted youth ≥85th percentile by WHO charts, post-hoc BMI categorisation identified a small subset near the cut-off that fell into normal weight on age/sex-specific recalculation. These cases were retained for comparative tables (Tables 1–5); sensitivity checks restricted to ≥85th percentile yielded the same conclusions.

**Table 1:** Respondents’ answers to the question “In those moments when you really ate too much, did you ever have the feeling that you could no longer control what and how much you ate?” taking into account BMI.

On those occasions when you have really eaten too much, have you ever had the feeling that you can no longer control what and how much you have eaten?	BMI							
	Underweight		Normal weight	Overweight	Class I obesity		Class II obesity	
	*n*	%	*n*	%	*n*	%	*n*	%
Yes	7	100%	37	84%	14	67%	12	71%
No	0	0%	7	16%	7	33%	5	29%
Chi-square	χ2 = 5,12			df = 3			p = 0,162	

n – number of participants; χ^2^ – Chi-square value; df – degrees of freedom; p – significance level; all tests two-tailed.

**Table 2:** Respondents’ answers to the question “Does your weight affect the way you think of yourself?” taking into account BMI.

Does your weight affect the way you think of yourself?	BMI							
	Underweight		Normal weight	Overweight	Class I obesity		Class II obesity	
	*n*	%	*n*	%	*n*	%	*n*	%
Never	1	14%	7	16%	2	10%	1	6%
Hardly ever	2	29%	6	14%	2	10%	2	12%
Very rarely	1	14%	4	9%	2	10%	3	18%
Rarely	0	0%	6	14%	1	5%	1	6%
Often	1	14%	9	20%	8	38%	4	24%
Very often	1	14%	7	16%	4	19%	1	6%
All the time	1	14%	5	11%	2	10%	5	29%
Chi-square	χ2 = 8,841			df = 15			p = 0,883	

n – number of participants; χ^2^ – Chi-square value; df – degrees of freedom; p – significance level; all tests two-tailed.

**Table 3:** Respondents’ answers to the question “Were you dissatisfied with your weight?” taking into account BMI.

Were you unhappy with your weight?	BMI							
	Underweight		Normal weight	Overweight	Class I obesity		Class II obesity	
	*n*	%	*n*	%	*n*	%	*n*	%
Never	1	14%	6	14%	1	5%	0	0%
Hardly ever	3	43%	7	16%	1	5%	1	6%
Very rarely	0	0%	0	0%	1	5%	2	12%
Rarely	1	14%	8	18%	0	0%	0	0%
Often	0	0%	9	20%	7	33%	5	29%
Very often	2	29%	12	27%	6	29%	4	24%
All the time	0	0%	2	5%	5	24%	5	29%
Chi-square	χ2 = 31,326			df = 18			p = 0,0261	

n – number of participants; χ^2^ – Chi-square value; df – degrees of freedom; p – significance level; all tests two-tailed.

**Table 4:** Respondents’ answers to the question “Were you afraid that others would see you eating?” taking BMI into account.

Were you afraid that others would see you eating?	BMI							
	Underweight		Normal weight	Overweight	Class I obesity		Class II obesity	
	*n*	%	*n*	%	*n*	%	*n*	%
Never	6	86%	22	50%	1	5%	6	35%
Hardly ever	0	0%	4	9%	1	5%	1	6%
Very rarely	0	0%	3	7%	0	0%	2	12%
Rarely	1	14%	3	7%	2	10%	1	6%
Often	0	0%	7	16%	6	29%	4	24%
Very often	0	0%	1	2%	8	38%	1	6%
All the time	0	0%	4	9%	3	14%	2	12%
Chi-square	χ2 = 21,574			df = 18			p = 0,0042	

n – number of participants; χ^2^ – Chi-square value; df – degrees of freedom; p – significance level; all tests two-tailed.

**Table 5:** Respondents’ answers to the question “Did you ever feel uncomfortable or shy about looking at your own body? For example in the mirror, in shop windows, when undressing, or when taking a bath or shower” including BMI.

Did you ever feel uncomfortable or shy about your own body? For example, in the mirror, in a shop window, when undressing or when taking a bath or shower.	BMI							
	Underweight		Normal weight	Overweight	Class I obesity		Class II obesity	
	*n*	%	*n*	%	*n*	%	*n*	%
Never	4	57%	11	25%	2	10%	3	18%
Hardly ever	1	14%	4	9%	2	10%	0	0%
Very rarely	0	0%	4	9%	4	19%	3	18%
Rarely	1	14%	6	14%	3	14%	1	6%
Often	0	0%	10	23%	5	24%	3	18%
Very often	0	0%	5	11%	1	5%	3	18%
All the time	1	14%	4	9%	4	19%	4	24%
Chi-square	χ2 = 16,716			df = 18			p = 0,535	

n – number of participants; χ^2^ – Chi-square value; df – degrees of freedom; p – significance level; all tests two-tailed.

Ultimately, 100 people (54 girls and 46 boys) were selected for the study, with an average age of 14.6 years (SD = 2.4). All 100 participants were included in the subsequent statistical analyses; no cases were excluded due to missing data. The participants came from both urban (51%) and rural areas (49%), which enabled an analysis of the impact of the environment on health behavior. Anthropometric measurements were taken in accordance with current standards – height was measured to the nearest 0.1 cm using a SECA 213 stadiometer, and body weight to the nearest 0.1 kg using a SECA 813 electronic scale. Based on the data obtained, BMI was calculated and compared to the WHO centile charts.

All participants completed a self-developed questionnaire covering sociodemographic information, eating habits, level of physical activity, sleep quality, body image and sources of motivation for weight reduction. In addition, semi-structured interviews were conducted with a subgroup of 50 participants whose answers indicated extremely high or low levels of motivation, in order to deepen the qualitative analysis.

### 2.3 Process

All measurements were carried out by a qualified and trained team of nutritionists with experience in working with children and young people and in conducting public health research. In order to collect empirical data, a set of standardized research tools was used to enable a comprehensive assessment of the factors influencing the motivation to lose weight and the lifestyle of the participants.

The main research tool was an original questionnaire developed on the basis of scientific literature and existing, previously verified tools in the field of public health and health psychology. The questionnaire included closed single- and multiple-choice questions, semi-open questions and statements rated on a five-point Likert scale. The questionnaire covered sociodemographic data (age, gender, place of residence, family’s financial status, parents’ education), lifestyle (eating habits, physical activity, sleep), perception of one’s own body weight, level of self-esteem, as well as sources of motivation and barriers in the process of losing weight.

In addition, anthropometric measurements were taken to supplement and verify the declarative data. Height was measured to the nearest 0.1 cm using a SECA 213 stadiometer, and body weight was measured to the nearest 0.1 kg using a SECA 813 electronic scale. The measurements were carried out in all three research settings (school, hospital, rehabilitation center), in light clothing and without shoes, in accordance with measurement standards - the measurements were taken twice and then the results were averaged. The body mass index (BMI) was calculated using the formula: body mass [kg] / (height [m])^2^ and interpreted according to WHO standards - overweight: BMI ≥ 85th percentile, obesity: BMI ≥ 95th percentile.

In order to enrich the quantitative data with qualitative context, semi-structured individual interviews were conducted with a selected group of participants (*n* = 50) who were found to have extremely high or low levels of motivation. These interviews focused on topics such as family and peer relationships, body image, emotional experiences and the perceived effectiveness of weight loss efforts. The combination of quantitative and qualitative methods enabled data triangulation and a more comprehensive understanding of the phenomenon under study.

### 2.4 Research questionnaire

The questionnaire contained closed questions with single and multiple choice, semi-open questions, and a set of statements rated on a five-point Likert scale, allowing the degree of agreement with the presented statements to be determined. It covered the following topics: sociodemographic data (age, gender, place of residence, family’s financial situation, parents’ level of education), lifestyle (daily eating habits, regularity of meals, level of physical activity, length and quality of sleep), health behavior (time spent in front of a screen, participation in sports, dieting), perception of one’s own body weight, level of self-esteem, and the main sources of motivation and barriers to taking health-promoting action.

The questionnaire was developed specifically for the purposes of this research but was not entirely custom-made. Its construction was based on a comprehensive review of the scientific literature and existing validated tools in health psychology and research on health behavior in children and adolescents. In particular, the Eating Disorder Examination Questionnaire (EDE-Q), validated and widely applied in Belgium, served as a conceptual and structural reference. Thus, the present study constitutes an initial step toward the cultural and linguistic adaptation of the EDE-Q for the Polish context. In addition, items were formulated to reflect constructs described in theoretical frameworks such as Self-Determination Theory and previous empirical findings on motivational and family-related factors in weight reduction.

Internal consistency of the scales in the present sample was satisfactory (Cronbach’s *α* = 0.72–0.84), which indicates acceptable reliability of the instrument. Nevertheless, as the Polish version of the questionnaire has not yet undergone full formal validation, this should be considered a methodological limitation, and the findings should be interpreted with caution.

### 2.5 Assessment of motivation to lose weight

Adapted elements of the Self-Determination Theory (SDT) developed by Deci and Ryan (6) were used to assess the level of motivation. The following motivations were examined:

- Internal (desire to improve health, well-being, appearance),
- External (pressure from parents, doctors, school environment),
- Amotivation (lack of willingness to take action).

The reference point was the self-assessment scale and the declared readiness to change (stages of change model). Social support (from parents, peers and school) was also assessed as a potential moderator of the level of motivation. Motivation was assessed in three dimensions: internal motivation, external motivation and amotivation. These were not single answer options, but thematic scales, each consisting of a set of statements rated on a five-point Likert scale (from “strongly disagree” to “strongly agree”). The final score for each dimension was calculated as the arithmetic mean of the relevant items, which allowed us to differentiate between the dominant type of motivation among participants. This approach ensured that motivation was treated as a continuous construct rather than a categorical variable.

Family support was assessed using a set of items completed by parents or legal guardians on behalf of their children. This approach allowed for a consistent and reliable description of the child’s family environment and parental involvement in the process of behavior change. The measure covered three dimensions: emotional (expressions of acceptance, understanding and encouragement), practical (joint meal preparation, shared eating and support for physical activity) and informational (providing knowledge and discussions about healthy eating and lifestyle). The overall family support score was calculated as the mean of all items, with higher values indicating stronger parental engagement in supporting the child. This parent-reported measure provided an objective reflection of the actual level of family involvement in promoting healthy behavior change.

### 2.6 Statistical analysis

All data collected during the study was subjected to statistical analysis using IBM SPSS Statistics version 27.0 and Microsoft Excel 365 spreadsheet. Descriptive statistics were analyzed first, aimed at characterizing the studied population in terms of sociodemographic variables, lifestyle, health attitudes and level of motivation. Arithmetic means, medians, standard deviations, minimum and maximum values, as well as the frequency of occurrence of individual characteristics were calculated. Categorical data were analyzed by determining percentage distributions.

In order to compare the results between groups (e.g., boys vs. girls, pupils from cities vs. villages, different BMI levels), appropriate statistical tests were used. For qualitative variables, Pearson’s chi-square test was used, while for quantitative variables meeting the criteria of normal distribution, the Student’s t-test for independent samples was used. In cases where the normality assumption was not met, non-parametric alternative tests were used, such as the Mann–Whitney U test.

In a further step, a logistic regression analysis was carried out to identify independent predictive factors for selected dependent variables, such as the level of motivation to lose weight (high vs. low) and the effectiveness of weight reduction (reduction vs. no reduction). Variables such as age, gender, level of physical activity, social support, self-esteem, time spent in front of a screen, emotional factors and place of residence were taken into account. The results of the regression analysis were presented in the form of odds ratios (OR) with 95% confidence intervals.

The reliability and internal consistency of the scales and sets of questions used to assess motivation and health attitudes were verified using Cronbach’s alpha coefficient. The obtained values ranged from 0.72 to 0.84, which indicates the high reliability and usefulness of the research tools. In the case of missing data (the percentage of which did not exceed 3% in any set of questions), the listwise deletion method was used, which allowed for consistency of analyses and avoided errors resulting from data imputation.

All statistical tests were performed with an accepted significance level of *α* = 0.05. The results were considered statistically significant if the *p*-value was less than 0.05. Where appropriate, the strength of the relationship (e.g., Cramér’s *V* or eta-squared) and effect size were also reported to better interpret the practical significance of the results.

In addition to descriptive statistics and chi-square tests, regression modelling was applied to account for the limited cell sizes in contingency tables. Logistic regression analyses were used to identify predictors of motivation and the effectiveness of weight reduction, treating motivation type and selected behavioral outcomes as dependent variables. This approach allowed for a more robust assessment of the relationships between family support, BMI, self-esteem and other variables. Chi-square tests were therefore used primarily for preliminary exploration of associations, whereas regression models provided the main basis for statistical inference.

## 3 Results

### 3.1 Characteristics of the test group

A total of 100 people aged between 10 and 18 years old took part in the study. The analyses presented in the following subsections were therefore carried out on the entire group of 100 participants. For the sake of clarity and accessibility of the presented data, the full wording of selected survey questions is repeated in the Results section. This approach was adopted deliberately to ensure that all readers, regardless of their familiarity with the questionnaire, can easily interpret the finding. They met the inclusion criteria, which included being diagnosed as overweight or obese according to the WHO classification (BMI above the 85th percentile for a given age and gender). It should be noted that in the present analysis BMI values were used as the primary anthropometric indicator. However, in pediatric populations, BMI z-scores or BMI percentiles are more appropriate measures as they account for age- and sex-specific developmental trajectories. These indices were not included in the current analysis but will be incorporated in future work to provide a more accurate assessment of weight status in children and adolescents.

The largest group consisted of 38 students from the Secondary School in Tyczyn (38% of the total sample). The other two groups were equal in terms of the number of respondents – 31 people each came from the Clinical Regional Hospital No. 2 in Rzeszów and the Zimowit Spa Hospital in Rymanów-Zdrój. The characteristics of the respondents according to the facility are presented in Table 6.

**Table 6:** Characteristics of respondents by site.

Parameter	Secondary school in Tyczyn	Clinical Regional Hospital No. 2 in Rzeszów	Zimowit Health Resort Hospital in Rymanów-Zdrój	Total/Overall average
Number of participants	38	31	31	100
Average age (years)	16,5	13,2	14,1	14,6
Number of girls	20	18	16	54
Number of boys	18	13	15	46
Number of people from cities	21	18	12	51
Number of people from villages	17	13	19	49
Average body weight (kg)	79,2	72,8	76,4	76,1
Average height (cm)	171,3	162,5	165,8	166,5
Average BMI (kg/m^2^)	26,9	27,6	27,8	27,4

The average age of the participants was 14.6 years (SD = 2.4). The youngest participants in the study were 10 years old (mainly in the group of hospital patients), while the oldest were 18 years old (in the high school group). Broken down by institution, the high school students were on average 16.5 years old, the patients in the hospital in Rzeszów 13.2 years old, and the people staying at the health resort 14.1 years old. These data show that the study covered key developmental periods, such as late childhood, adolescence and early youth, which are particularly important in shaping eating habits and attitudes towards health.

Fifty-four girls (54%) and 46 boys (46%) took part in the study. The gender ratio was relatively balanced in all facilities, which enabled intergroup comparisons without significant distortions due to unequal numbers. Girls slightly dominated the hospital and spa group, while the high school had an almost equal proportion of both genders.

The respondents came almost equally from urban (51%) and rural (49%) areas, which allowed for an analysis of the impact of the living environment on eating habits, the level of physical activity and the perception of the need to change body weight. The group of high school students was dominated by city dwellers, while in the spa town there was a noticeable predominance of patients from rural areas, which may be due to the profiles of the children referred there.

The average body weight of the subjects was 76.1 kg, with a minimum value of 58.5 kg and a maximum value of 103.4 kg. The highest average body weight was recorded among high school students (79.2 kg), which may be related to their more advanced age and puberty. In contrast, the lowest average body weight was recorded for hospitalized children at 72.8 kg.

The average height of the participants was 166.5 cm, with the tallest being high school students (171.3 cm) and the shortest being patients at the hospital in Rzeszów (162.5 cm), which is consistent with the age of the participants.

The average BMI for the whole group was 27.4 kg/m^2^, which qualifies as overweight according to WHO standards, bordering on obesity. In the individual groups, the BMI values were slightly higher in the medical facilities than in the secondary school, which may be related to the more severe severity of the problem among children referred for treatment or rehabilitation. The highest average BMI was recorded in the spa group - 27.8, which may indicate the presence of cases of morbid obesity.

### 3.2 Respondents’ answers

When asked, ‘Have you ever tried to eat less to change your figure or lose weight?’ the respondents gave a variety of answers, which can be analyzed in detail both numerically and in percentage terms. The largest group, 27 people (34.18%), answered that they had not tried to eat less at all. This means that more than a third of the respondents did not take any dietary measures to change their figure or weight. This may suggest a lack of awareness of the problem, lack of motivation, or a preference for methods other than reducing the amount of food consumed. Another significant group are people who have tried to restrict their food intake for 1–5 days – 22 respondents (27.85%). This result suggests that almost one third of the respondents made attempts to reduce their food intake, but these were short-term interventions that probably did not have lasting effects. Thirteen people (16.46%) declared that they tried to eat less for 6–12 days. This indicates that some respondents made moderate efforts to change their figure, but these attempts were also not very long-lasting, which may indicate difficulties in maintaining motivation. The group that restricted food for 13–15 days consisted of 15 people (18.99%). Compared to previous results, these people took more decisive action towards changing their diet, which may suggest greater commitment to the weight loss process. Ten respondents (12.66%) tried to limit food intake for 16–22 days, which shows even greater consistency in their actions. Although they represent a smaller part of the surveyed group, it can be seen that these efforts were more persistent. It is worth noting that none of the respondents (0%) declared that they had tried to restrict food for 23–27 days, which indicates a clear break in the number of days of the attempts.

**Figure 1:**
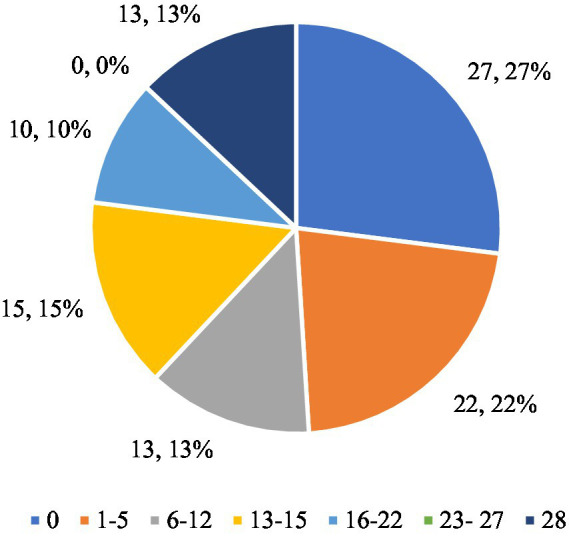
Respondents’ answers to the question ‘Have you ever tried to eat less to change your body shape or weight?’.

Among the participants, 13 individuals (16.46%) declared that they had restricted food intake for a full 28 days, which represented the longest period of using this strategy within the studied group and indicates a relatively high level of consistency in their actions. The majority of respondents (around 66%) have attempted to restrict their food intake, however, these were mostly short-term interventions (up to 15 days). Only 16.46% of respondents consistently restricted their food intake for a whole month, which indicates the difficulty of maintaining a long-term diet. These results show that although there is motivation to reduce food intake in order to change one’s body shape, it is often short-lived and inconsistent. Figure 1 shows the respondents’ indications regarding the question ‘Have you tried to eat less to change your body shape or weight?’

**Figure 2:**
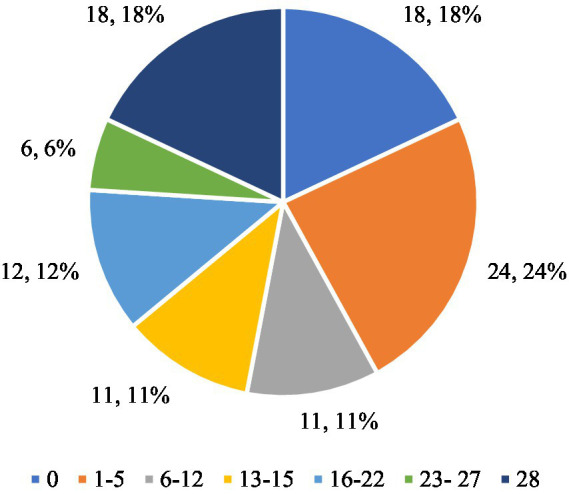
Respondents’ answers to the question ‘Have you ever tried to follow a certain diet to change your weight or body shape? For example, a certain amount of food, a certain number of calories, or rules about what and when you had to eat’.

The analysis of the respondents’ answers to the question “Have you tried to follow certain dietary rules to change your weight or body shape? For example, a certain amount of food, a certain number of calories, or rules about what and when you had to eat” provides information on the respondents’ attempts to follow dietary rules. The respondents’ answers to the question ‘Have you tried to follow certain dietary rules to change your weight or body shape? For example, a certain amount of food, a certain number of calories, or rules about what and when you had to eat.’ They are presented in Figure 2. The results allow for numerical and percentage analysis. Eighteen respondents (22.78%) stated that they had not tried to follow any dietary rules (0 days). This is a relatively small proportion of the respondents, which indicates that the majority of people took measures to follow certain dietary rules in order to change their weight or figure. This means that a large proportion of respondents were aware of the need to change their eating habits. Twenty-four people (30.38%) stated that they had tried to follow dietary rules for 1–5 days. This is the largest group, which suggests that a significant proportion of respondents made short-term attempts to introduce dietary rules, but lacked the consistency to continue them for a longer period of time. Eleven people (13.92%) followed the nutritional rules for 6–12 days. This result indicates a moderate commitment of this group to the application of nutritional rules, although these were not long-term attempts. Eleven respondents (13.92%) declared that they had followed the rules for 13–15 days, which indicates a similar number of people as in the group following the rules for 6–12 days. This means that a certain proportion of the respondents made more consistent attempts to change their eating habits. Twelve respondents (15.19%) tried to follow the rules for 16–22 days, which indicates greater consistency in adhering to the nutritional rules than for shorter periods. Six people (7.59%) declared that they followed the rules for 23–27 days. This is a relatively small group, but it shows that some people managed to persevere for a longer period of time. Eighteen people (22.78%) answered that they followed the nutritional rules for a full 28 days, which is the second largest group after people who followed the rules for 1–5 days. This result indicates that almost 23% of the respondents were able to consistently follow certain dietary rules for a whole month, which proves their motivation and commitment to the process of changing their body shape.

The largest proportion of respondents (30.38%) tried to follow the nutritional rules for a short period (1–5 days), which may indicate initial motivation that quickly faded. However, a similarly large group (22.78%) was able to maintain the dietary rules for a full 28 days, which shows that some respondents were very consistent and motivated to change their eating habits. The other groups that declared adherence to the rules for longer periods (6–12, 13–15, 16–22 days) together make up about 42.03% of the respondents, which suggests that many people took action but had difficulty maintaining it for a whole month. The 7.59% of respondents who persevered for 23–27 days indicates that a small group was very close to fully implementing the rules, but for various reasons was unable to persevere to the end. Overall, the data shows that most of the participants tried to follow the nutritional rules, but only a small group was motivated enough to follow these rules for a longer period of time.

**Figure 3:**
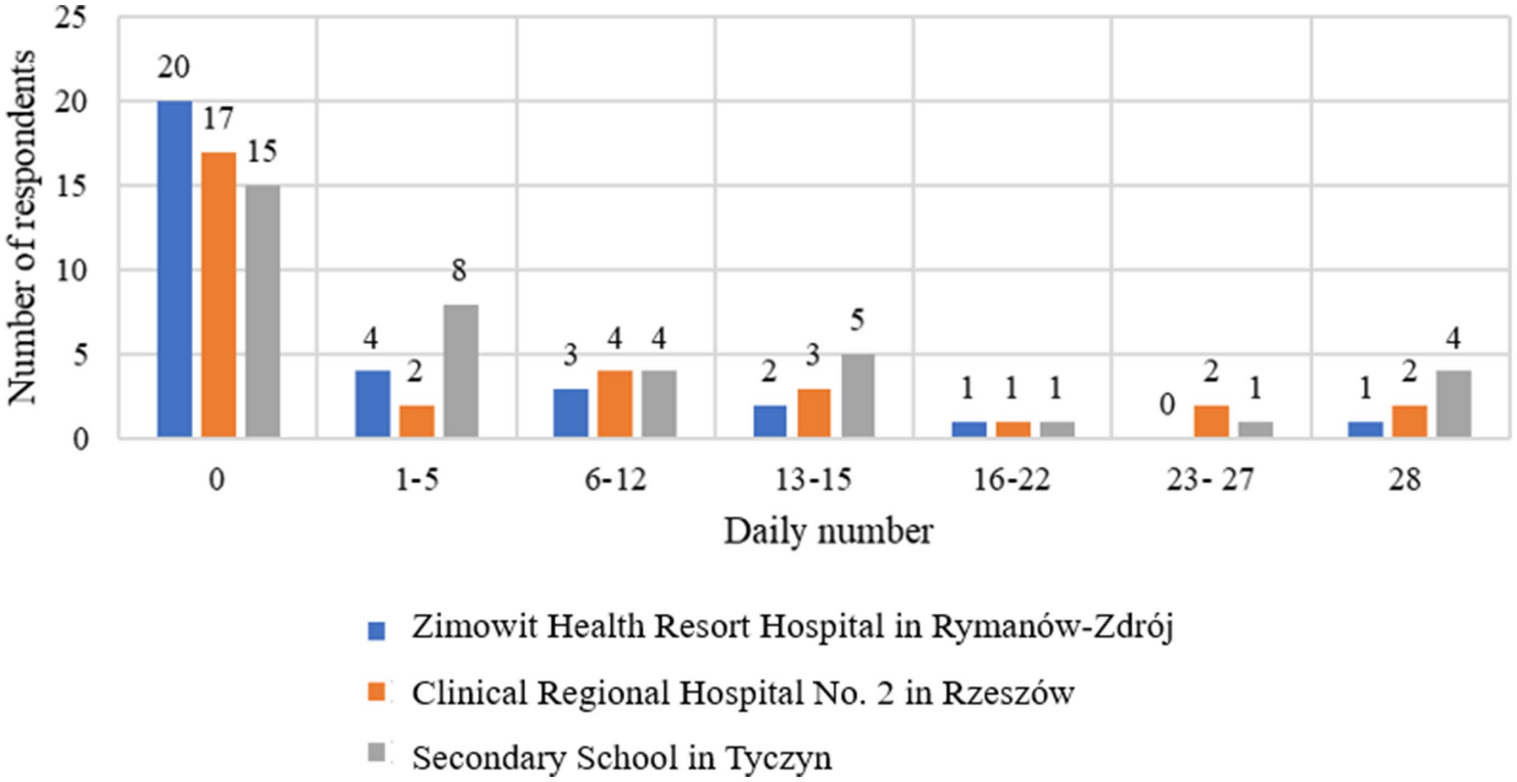
Respondents’ indications regarding the question “Were you afraid that you would lose control over your eating behavior?” broken down by survey location.

Figure 3 shows the respondents’ answers to the question “Were you afraid of losing control over your eating behavior?” broken down into three survey locations: the Zimowit Health Resort Hospital in Rymanów-Zdrój, the Clinical Regional Hospital No. 2 in Rzeszów and the Secondary School in Tyczyn. The analysis of the results shows differences in the perceived fears of losing control over eating in individual groups. In the Zimowit Health Resort Hospital in Rymanów-Zdrój, 20 respondents (58.82%) declared that they were not afraid of losing control over their eating behavior (0 days). This means that the majority of patients felt confident in managing their eating habits and had no serious fears of losing control. Four people (11.76%) feared losing control for 1–5 days, which indicates occasional, short-term fears. Three people (8.82%) had these fears for 6–12 days, and two people (5.88%) stated that they had these fears for 13–15 days, which indicates longer but shorter episodes of anxiety. One person (2.94%) feared losing control for 16–22 days, and one person (2.94%) struggled with these fears for a full 28 days.

At the Clinical Regional Hospital No. 2 in Rzeszów, 17 respondents (53.13%) declared that they did not fear losing control over their eating behavior (0 days), which suggests that more than half of the patients from Rzeszów did not have significant fears related to losing control over eating. Two people (6.25%) were afraid for 1–5 days and four people (12.50%) for 6–12 days, which suggests moderate anxiety in this group. Three people (9.38%) had fears for 13–15 days, and one person (3.13%) struggled with fears for 16–22 days. Two people (6.25%) had fears for 23–27 days, and two people (6.25%) for a full 28 days, which indicates more intense anxiety in some patients.

At the Secondary School in Tyczyn, 15 respondents (40.54%) declared that they were not afraid of losing control over their eating behavior (0 days). This is the lowest percentage among the surveyed groups, which suggests that the majority of students had some concerns about controlling their eating habits. Eight people (21.62%) had concerns for 1–5 days, and 4 people (10.81%) for 6–12 days, which indicates moderate anxiety in this group. Five people (13.51%) had concerns for 13–15 days, which indicates more frequent anxiety among students. One person (2.70%) feared losing control for 16–22 days, and one person (2.70%) had concerns for 23–27 days. Four people (10.81%) declared that they feared losing control for a full 28 days, which is the highest percentage in this category among all groups.

The highest number of people who were not afraid of losing control over their eating behavior came from the Zimowit Health Resort Hospital in Rymanów-Zdrój (58.82%) and the Clinical Regional Hospital No. 2 in Rzeszów (53.13%), which suggests that patients from these facilities were less likely to experience fears of losing control over their eating. At the Secondary School in Tyczyn, only 40.54% of students did not have such fears, which suggests that students were more likely to experience fears of losing control over their eating behavior. The highest number of people who feared losing control for a full 28 days came from Tyczyn (10.81%), which indicates that the students at this school more often struggled with intense fears regarding their eating habits compared to hospital patients, where such fears were less common.

**Figure 4:**
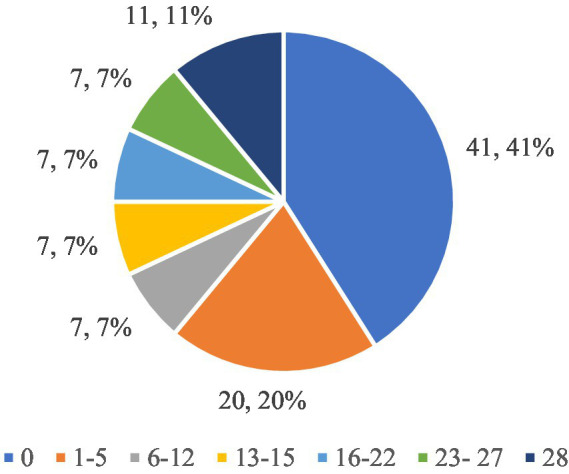
Respondents’ answers to the question ‘How often have you felt guilty after eating in the last 4 weeks? So much so that you feel you have done something wrong’.

Figure 4 shows the respondents’ answers to the question “How often in the last 4 weeks have you felt guilty after eating? So much so that you felt you had done something wrong,” which allows us to assess how often the respondents felt guilty after eating. Forty-one respondents (51.90%) stated that they never felt guilty after eating (0 days). This is the largest group, which suggests that for the majority of respondents, eating did not involve feelings of guilt or a sense of having done something wrong. This means that food was not a source of emotional difficulties for this group. Twenty people (25.32%) admitted that they felt guilty for 1–5 days, which means that for this group guilt occurred occasionally and was not a regular problem. These people had such thoughts rarely and they were not a dominant element of their eating experiences. 7 respondents (8.86%) admitted to feeling guilty for 6–12 days, which suggests that for this group, guilt after eating was a more noticeable problem, although not an everyday one. Seven people (8.86%) said they felt guilty for 13–15 days, which means that these thoughts were more regular and occurred for almost half a month. Seven people (8.86%) admitted to feeling guilty for 16–22 days, which indicates that for this group, feeling guilty after eating was a problem lasting for most of the month. Seven people (8.86%) stated that they felt guilty for 23–27 days, which means that for this group, feeling guilty after eating was an almost daily problem. The largest group after those who never felt guilty is made up of 11 people (13.92%) who admitted that they felt guilty after eating every day for a full 28 days. This means that for this group, eating caused a constant feeling of guilt, which may indicate more serious emotional problems related to eating and the perception of their weight.

The largest proportion of respondents (51.90%) never felt guilty after eating, suggesting that food was not a source of stress or a sense of doing something wrong for them. However, 13.92% of respondents felt guilty after eating every day for a full 28 days, indicating strong and regular emotional difficulties related to food. The remaining people felt guilty with varying frequency (1–27 days), which suggests that for many respondents, eating was associated with guilt, albeit to varying degrees and with varying intensity.

**Figure 5:**
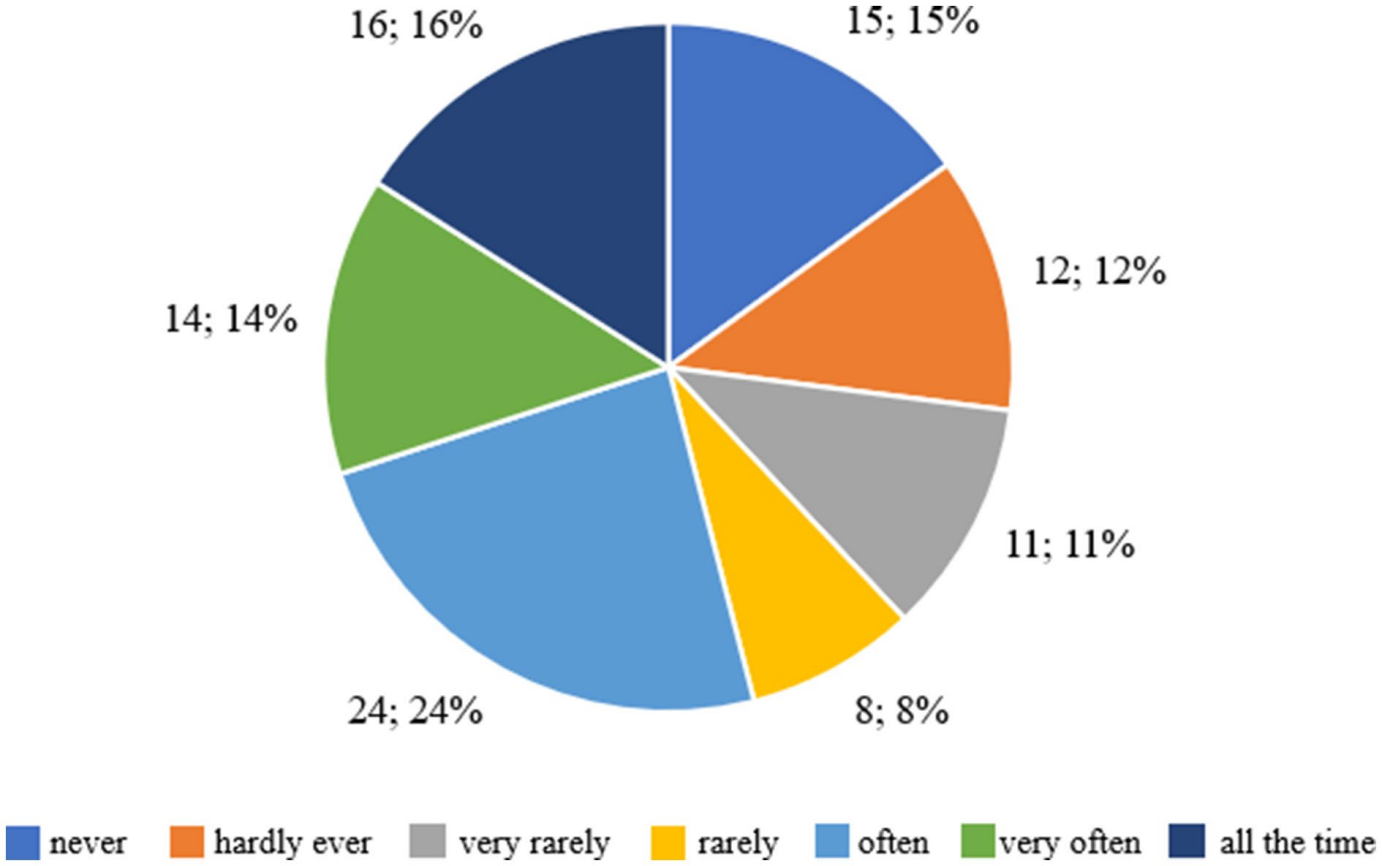
Respondents’ answers to the question ‘Does your weight affect the way you think of yourself?’.

Figure 5 shows the respondents’ answers to the question “Does your weight influence the way you think of yourself?,” which allows us to assess how often body weight affects respondents’ self-esteem and thoughts about themselves. Fifteen respondents (19%) answered that their weight never influences how they think of themselves, which means that for this group, weight does not play a role in their self-perception. Twelve people (15%) admitted that weight almost never affects their self-esteem, which means that weight has little impact on how they think of themselves. Eleven respondents (13.92%) stated that weight very rarely affects their self-image, which suggests that although weight does play a role in their self-esteem, it is not a frequent one. Eight people (10.13%) answered that weight rarely influences their thoughts, which means a moderate impact of weight on self-perception.

However, 24 people (30.38%) admitted that weight often influences the way they think about themselves, which means that for this group, weight is an important element of their self-esteem. Fourteen respondents (17.72%) stated that weight very often influences their thoughts, which indicates that weight has a regular and significant impact on their self-esteem. Furthermore, 16 people (20.25%) admitted that weight influences their self-perception all the time, which means that for this group, weight is the dominant factor shaping their self-esteem.

The majority of respondents (67.72%) stated that weight affects their thoughts about themselves to varying degrees – from ‘often’ to ‘all the time’. This means that for many respondents, body weight plays an important role in shaping their self-esteem. On the other hand, 32.28% of respondents declared that weight affects their self-image rarely or never, which indicates that for this group, weight is not a key element in the context of their self-perception.

**Figure 6:**
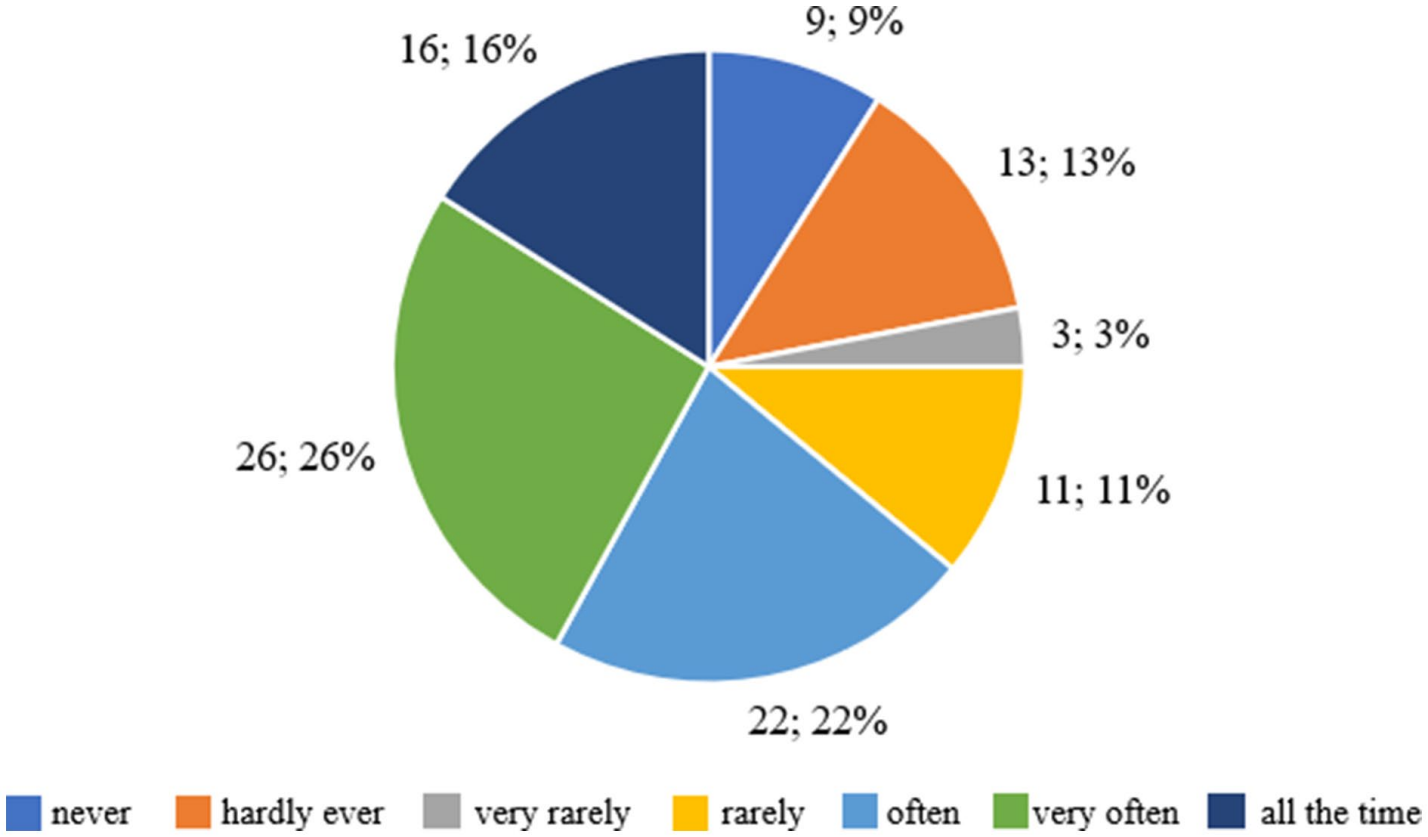
Respondents’ answers to the question ‘Would you have no problem if you were asked to weigh yourself once a week for the next month?’.

Figure 6 shows the respondents’ answers to the question “Would you have no problem if you were asked to weigh yourself once a week for the next month?,” which allows us to assess how the respondents perceive regular weighing and any possible discomfort associated with it. Nine respondents (11.39%) stated that they would never have a problem with weighing themselves regularly once a week for a month, which means that for this group, weight control would not cause any difficulties. Thirteen people (16.46%) admitted that they would almost never have a problem with it, which suggests that weighing would only cause occasional discomfort. Three respondents (3.80%) stated that they would very rarely have difficulties with weighing, which means that the impact of this activity would be minimal. Eleven people (13.92%) declared that they would rarely have a problem with regular weighing, which indicates a moderate level of discomfort. Twenty-two people (27.85%) admitted that they would often have difficulties with regular weighing, which suggests that for this group, weekly weighing would cause significant stress. Twenty-six respondents (32.91%) declared that they would very often have a problem with weighing themselves, which indicates that they would regularly struggle with discomfort during this activity. Finally, 16 people (20.25%) stated that they would have a problem weighing themselves once a week for a month, which means that for this group, regular weighing would be a constant source of stress and discomfort.

The majority of respondents (72.78%) admitted that regular weighing would make them feel uncomfortable to varying degrees – from ‘often’ to ‘all the time’. This means that many of the respondents would find it difficult to monitor their weight regularly. On the other hand, 27.22% of the respondents stated that weighing would not be a problem for them or would cause only slight discomfort.

**Figure 7:**
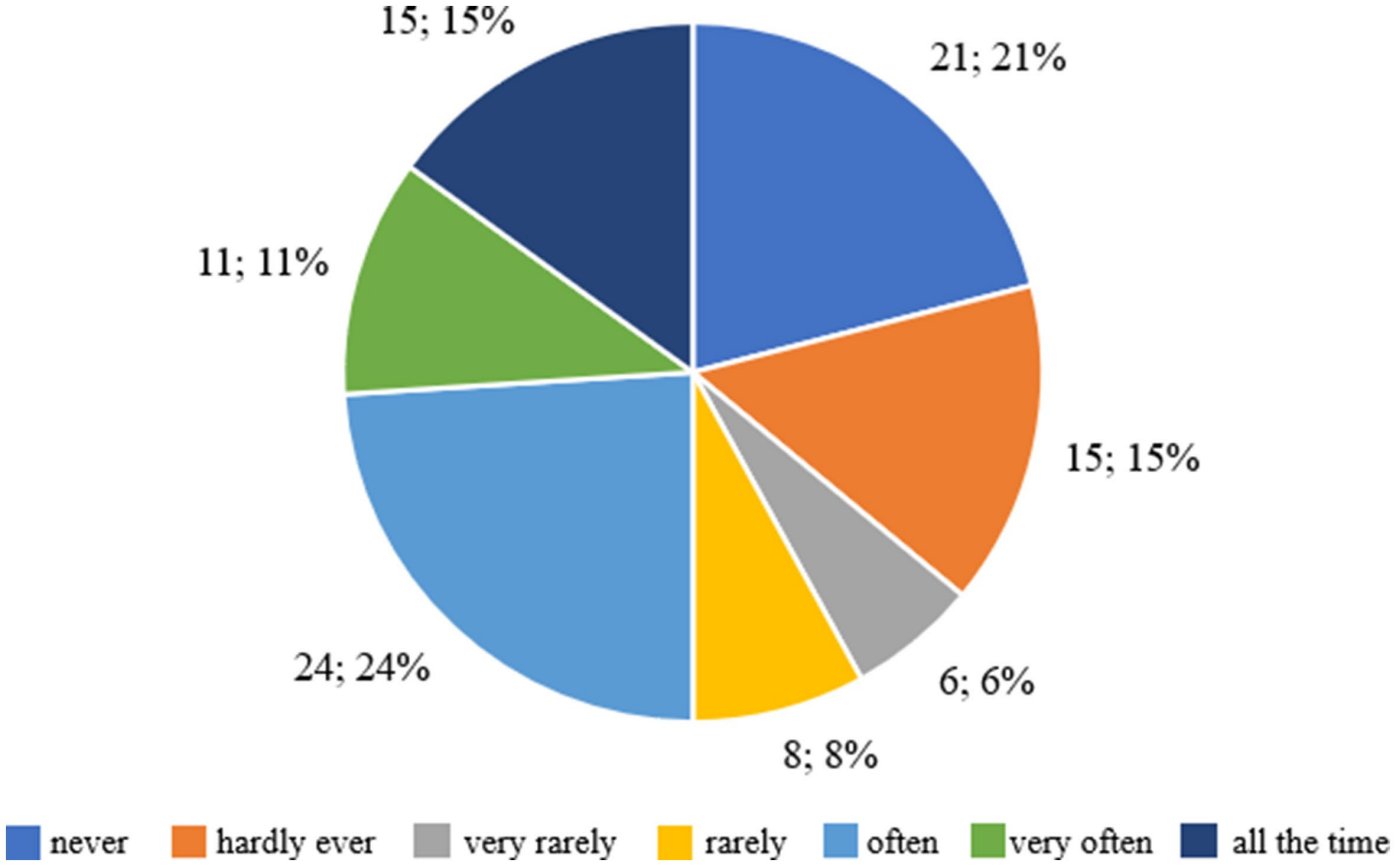
Respondents’ answers to the question ‘Did you feel uncomfortable or shy when others could see your body? For example, when changing in shared changing rooms, when swimming’.

Figure 7 shows the respondents’ answers to the question about the level of discomfort associated with the perception of their own body in various situations, such as looking at themselves in the mirror, while bathing or undressing. Twenty-one respondents (26.58%) declared that they had never felt uncomfortable or shy about seeing their own body. This means that for this group, the appearance of the body does not cause negative emotions or problems with self-acceptance. Fifteen people (18.99%) admitted that they almost never had such feelings, which suggests that occasional situations of uncertainty may occur, but in general the appearance of the body is not a source of discomfort for them. Six respondents (7.59%) stated that they very rarely felt uncomfortable with their body, which means that negative feelings occurred rarely and were of little importance. Eight people (10.13%) stated that they rarely felt uncomfortable with their body image, which suggests moderate problems with accepting their appearance. Twenty-four respondents (30.38%) admitted that they often felt uncomfortable or shy, which indicates clear difficulties with accepting their own body in this group. Eleven people (13.92%) stated that they had these feelings very often, and 15 respondents (18.99%) said that they felt uncomfortable all the time, which means that for this group, the perception of their own body was a constant source of uncertainty and discomfort.

The majority of respondents, 63.29%, felt discomfort related to the perception of their body to varying degrees - from ‘often’ to ‘all the time’. This suggests that for a significant proportion of respondents, body image is a source of negative emotions and difficulties in accepting themselves. On the other hand, 36.71% of respondents said that such feelings occurred rarely or not at all, which means that for this group, body image was not a cause for insecurity.

### 3.3 Statistical analysis of the respondents’ indications

In order to analyze the impact of the body mass index (BMI) on various aspects related to food control, physical activity and self-perception, the BMI was calculated for all respondents. The study examined how BMI affects responses regarding: difficulty in controlling the amount of food consumed at times when the respondent felt that they had eaten too much; the frequency of feeling a lack of control over eating in the last 4 weeks; the intensity of physical exercise in the last 4 weeks to avoid gaining weight; the impact of weight on the way they think about themselves; the impact of body shape on the way they think about themselves; the comfort of regularly weighing oneself over the next month; the level of dissatisfaction with one’s own weight; the level of dissatisfaction with one’s body shape; the fear that others might see one eating; and the comfort or shyness of seeing one’s own body in different situations, such as in a mirror, in a shop window, while bathing or changing. For each of these issues, statistical analyses were carried out using the chi-square test, which was calculated using a statistical program. The purpose of these analyses was to investigate whether there are significant differences in responses depending on the BMI category and to understand how different BMI values affect the perception of one’s own body and behaviors related to food control and physical activity.

Table 1 shows the responses regarding the feeling of losing control over the amount of food depending on BMI. The analysis shows that in the group of underweight people, all respondents (100%) felt a loss of control when they ate too much. In the group of people with a normal weight, 84% answered ‘YES’ and 16% ‘NO’. In the overweight group, 67% of respondents felt a loss of control, while 33% did not. In the groups with obesity grades I and II, 71% of the people in both groups felt a loss of control, and 29% did not. It should be noted that 3 people with grade III obesity were assigned to the grade II obesity group because the size of the individual groups was too small to perform a statistical analysis. The results of the Chi-square test showed a value of χ^2^ = 5.12 with df = 3 and *p* = 0.162. The *p*-value is greater than the standard significance level of 0.05, which means that the differences in the perception of loss of control over food intake depending on BMI are not statistically significant. This means that although there are differences in the proportions of people experiencing a loss of control in different BMI groups, they are not strong enough to be considered statistically significant.

In the underweight group, the distribution of answers was as follows: 14% answered ‘never’, 29% ‘almost never’, 14% ‘very rarely’, 0% ‘rarely’, 14% ‘often’, 14% ‘very often’ and 14% ‘all the time’. In the group with a normal weight, 16% answered ‘never’, 14% ‘almost never’, 9% ‘very rarely’, 14% ‘rarely’, 20% ‘often’, 16% ‘very often’ and 11% ‘all the time’. In the overweight group, 10% answered ‘never’, 10% ‘almost never’, 10% ‘very rarely’, 5% ‘rarely’, 38% ‘often’, 19% ‘very often’ and 10% ‘all the time’. In the group with grade I obesity, 6% answered ‘never’, 12% ‘almost never’, 18% ‘very rarely’, 6% ‘rarely’, 24% ‘often’, 6% ‘very often’ and 29% ‘all the time’. Similarly, in the group with grade II obesity, 6% answered ‘never’, 12% ‘almost never’, 18% ‘very rarely’, 6% ‘rarely’, 24% ‘often’, 6% ‘very often’, and 29% ‘all the time’. The Chi-square test showed a value of χ^2^ = 8.841 with df = 15 and *p* = 0.883. The *p*-value is greater than the standard significance level of 0.05, which means that the differences in the impact of weight on the way of thinking about oneself depending on BMI are not statistically significant. Despite the noticeable differences in responses between BMI groups, there is insufficient evidence that BMI has a significant impact on the way people think about themselves in relation to their weight.

Table 3 shows the responses to the question about dissatisfaction with one’s weight, taking into account different BMI categories. In the underweight group, 14% of respondents stated that they were ‘never’ dissatisfied with their weight, 43% answered ‘almost never’, 0% ‘very rarely’, 14% ‘rarely’, 0% ‘often’, 29% ‘very often’, and 0% ‘all the time’. In the group of people with a normal weight, 14% answered ‘never’, 16% ‘almost never’, 0% ‘very rarely’, 18% ‘rarely’, 20% ‘often’, 27% ‘very often’, and 5% ‘all the time’. The highest percentage of answers in this group was ‘very often’ and ‘often’. In the group of overweight people, 5% of respondents answered ‘never’, 5% ‘almost never’, 5% ‘very rarely’, 0% ‘rarely’, 33% ‘often’, 29% ‘very often’ and 24% ‘all the time’. In this group, the answers ‘often’, ‘very often’ and ‘all the time’ are dominant. In the group with grade 1 obesity, 0% answered ‘never’, 6% ‘almost never’, 12% ‘very rarely’, 0% ‘rarely’, 29% ‘often’, 24% ‘very often’, and 29% ‘all the time’. The majority of people in this group chose the answers ‘often’, ‘very often’ and ‘all the time’. In the group with grade II obesity, 0% answered ‘never’, 6% ‘almost never’, 12% ‘very rarely’, 0% ‘rarely’, 29% ‘often’, 24% ‘very often’ and 29% ‘all the time’. In this group, the answers ‘often’, ‘very often’ and ‘all the time’ are also dominant.

The Chi-square test showed a value of χ^2^ = 31.326 with df = 18 and *p* = 0.0261. The *p*-value is less than the standard significance level of 0.05, indicating statistically significant differences in dissatisfaction with one’s weight depending on BMI. This means that the level of dissatisfaction with weight varies significantly depending on the BMI category.

Table 4 shows the answers to the question about the fear of others seeing the respondent eating, broken down into different BMI categories. In the underweight group, 86% of respondents said they were ‘never’ they were worried about others seeing them eat, 0% answered ‘almost never’, 0% ‘very rarely’, 14% ‘rarely’, 0% ‘often’, 0% ‘very often’, and 0% ‘all the time’. In the group of people with a normal weight, 50% answered ‘never’, 9% ‘almost never’, 7% ‘very rarely’, 7% ‘rarely’, 16% ‘often’, 2% ‘very often’, and 9% ‘all the time’. In the group of overweight people, 5% of the respondents answered ‘never’, 5% ‘almost never’, 0% ‘very rarely’, 10% ‘rarely’, 29% ‘often’, 38% ‘very often’ and 14% ‘all the time’. In the group with grade I obesity, 35% answered ‘never’, 6% ‘almost never’, 12% ‘very rarely’, 6% ‘rarely’, 24% ‘often’, 6% ‘very often’, and 12% ‘all the time’. In the group with grade II obesity, 35% answered ‘never’, 6% ‘almost never’, 12% ‘very rarely’, 6% ‘rarely’, 24% ‘often’, 6% ‘very often’, and 12% ‘all the time’.

The Chi-square test showed a value of χ^2^ = 21.574 with df = 18 and *p* = 0.0042. The *p*-value is less than the standard significance level of 0.05, indicating that the differences in concerns about whether others will see the respondent eating, depending on BMI, are statistically significant. This means that the level of concern about eating in front of others varies significantly depending on the BMI category.

Table 5 shows the answers to the question about comfort and confidence with one’s own body, taking into account the different BMI categories. In the underweight group, 57% of respondents said they ‘never’ never felt uncomfortable or shy about their body, 14% answered ‘almost never’, 0% ‘very rarely’, 14% ‘rarely’, 0% ‘often’, 0% ‘very often’, and 14% ‘all the time’. In the group of people with a normal weight, 25% answered ‘never’, 9% ‘almost never’, 9% ‘very rarely’, 14% ‘rarely’, 23% ‘often’, 11% ‘very often’, and 9% ‘all the time’. In the group of overweight people, 10% of respondents answered ‘never’, 10% ‘almost never’, 19% ‘very rarely’, 14% ‘rarely’, 24% ‘often’, 5% ‘very often’ and 19% ‘all the time’. In the group with grade I obesity, 18% answered ‘never’, 0% ‘almost never’, 18% ‘very rarely’, 6% ‘rarely’, 18% ‘often’, 18% ‘very often’, and 24% ‘all the time’. In the group with grade 2 obesity, 18% answered ‘never’, 0% ‘almost never’, 18% ‘very rarely’, 6% ‘rarely’, 18% ‘often’, 18% ‘very often’, and 24% ‘all the time’. The Chi-square test showed a value of χ^2^ = 16.716 with df = 18 and *p* = 0.535. The *p*-value is greater than the standard significance level of 0.05, which means that the differences in perceived comfort and body confidence according to BMI are not statistically significant. This means that there are no significant differences in the level of discomfort or shyness associated with one’s own body depending on the BMI category.

Due to the relatively small cell sizes in some of the cross-tabulations, chi-square tests were applied only as an exploratory tool. To strengthen statistical inference, logistic regression models were additionally estimated, which allowed for identifying predictors of motivation and weight-related behaviors. Future studies with larger samples will make it possible to apply more advanced modelling strategies (e.g., multilevel regression, structural equation modelling).

To sum up, it should be noted that the study assessed the types of motivation to change eating behavior on the basis of selected items of the author’s questionnaire developed on the basis of the assumptions of the Self-Determination Theory (SDT). According to this concept, three main types of motivation are distinguished: autonomous (internal), controlled (external) and amotivation, understood as a lack of readiness to act. The analyzed questions made it possible to determine to what extent decisions regarding weight loss and maintaining healthy eating habits resulted from an internal need for change, and to what extent they were the result of environmental pressure or negative emotions related to body weight. Controlled motivation indicators were considered to be items in which the aim of the action was primarily to improve appearance, reduce body weight or avoid negative evaluation. Questions about trying to eat less or adhering to certain dietary rules reflected externally controlled behaviors, resulting from the need to obtain approval or fear of criticism. This group also included questions related to the fear of losing control over food and the readiness to weigh yourself regularly. The high level of anxiety or discomfort that accompanies these activities indicates the advantage of external regulation over autonomic regulation, in which emotions and social pressure dominate instead of a sense of agency.

Autonomous motivation includes actions taken of one’s own free will, resulting from the need for self-development, health and well-being. In this study, it was manifested mainly in questions related to adherence to dietary rules, which for some participants could be an expression of a conscious, planned approach to the process of weight reduction. Motivation understood in this way reflects an internal belief in the meaning of the actions taken, but in the analyzed group it had a limited scope and was often displaced by controlled motives.

Amotivation, i.e., the lack of an inner sense of influence and meaning of the efforts made, was revealed in questions about the feeling of guilt after eating and the belief that there is no control over one’s own eating behavior. Such emotional reactions indicate a low level of internal regulation and can lead to the perpetuation of a negative cycle of restrictions, frustration and re-breaking the rules.

The answers obtained indicate that in the studied group of overweight and obese children and adolescents, controlled motivation prevailed, i.e., an externally conditioned need to change appearance and avoid negative emotions. Elements of autonomic motivation appeared less frequently and were associated with planned attempts to modify lifestyle. The co-occurrence of amotivation in the form of guilt and helplessness in the face of food, on the other hand, explained the short-term nature of the actions taken and the difficulty in maintaining new habits. The profile of the obtained results confirms that an effective intervention in the field of weight reduction in children requires not only dietary control, but above all strengthening intrinsic motivation and emotional support from the family.

**Figure 8:**
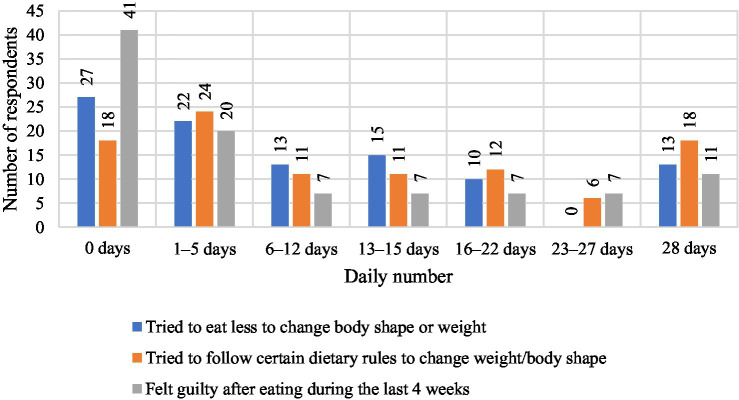
Summary of results on eating behavior and emotional responses in the last 28 days.

Figure 8 shows the distribution of responses for three variables assessed in terms of the number of days over the past 28 days, including attempts to eat less to change weight or figure, adherence to specific dietary rules, and frequency of experiencing guilt after eating. In the case of attempts to restrict food, 27 people, 27%, did not take any action, 22 people, 22%, declared this behavior for 1–5 days, and only 13 people, 13%, maintained it for the entire month. Similar trends were recorded for dietary adherence – 18 people, 18%, did not follow any rules, 24 people, 24%, followed them for a short time, and only 18 people, 18%, maintained them for 28 days. These data indicate that the majority of participants attempted change only occasionally and for a short time, reflecting the dominance of controlled motivation, based on external pressure and the need for a quick effect. With regard to the emotions that accompany food, 41 people, 41%, never felt guilt after eating a meal, 20 people, 20%, experienced it occasionally, and 11 people, 11%, experienced it daily. A total of 59% of respondents admitted that the feeling of guilt appeared at least once a month, which indicates the presence of amotivation, i.e., a lack of internal regulation and a reduced sense of agency.

To sum up, the data confirm that in the studied group of overweight and obese children and adolescents, controlled motivation, characterized by unstable and short-term involvement, prevailed. Autonomic motivation, based on the inner need for change and care for health, appeared rarely, while the presence of amotivation in the form of guilt additionally weakened the durability of the actions taken. These results indicate the need to strengthen intrinsic motivation and emotional and family support, which can be conducive to the long-term maintenance of health-promoting habits.

**Figure 9:**
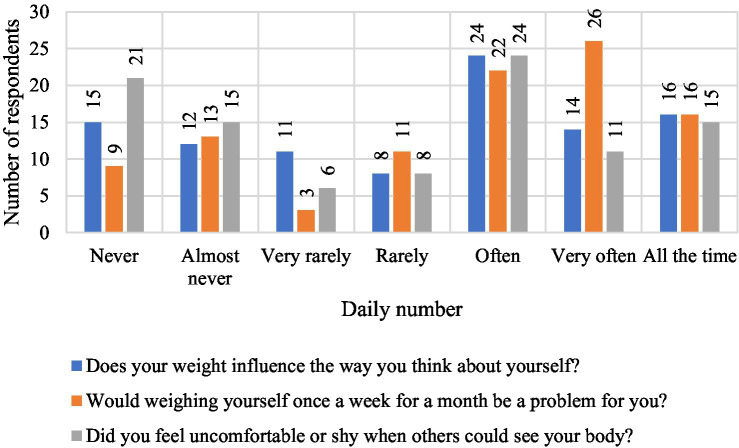
Summary of results concerning self-assessment, perceived discomfort during weighing and body perception among the children and adolescents surveyed.

Figure 9 shows the distribution of responses for three variables assessed on the seven-point Likert scale, including the effect of body weight on the way you think about yourself, the discomfort associated with regular weighing, and the feeling of shyness in situations where others could see the respondent’s body. When asked about the impact of weight on self-esteem, 15 people, 15%, indicated that weight never affects the way they think about themselves, while 24 people, 24%, said it happens often, 14 people, 14%, very often, and 16 people, 16%, all the time. In total, more than half of the respondents, 54%, felt the impact of body weight on self-esteem, indicating a strong presence of controlled motivation dependent on external evaluation.

When asked about weighing themselves, 9% said it would not be a problem for them, while 64% said they had varying degrees of discomfort, with 26% saying very often and 16% all the time. These results confirm that weight control was associated with negative emotions and tension, typical of extrinsic motivation. When asked about feeling shyness about their own body, 21 people, 21%, had never experienced this condition, while 50% of respondents indicated the answers “often” or “very often.” This means that half of the respondents felt discomfort related to body exposure, which also confirms the dominance of controlled motivation of a social nature.

To sum up, the data confirm the predominance of extrinsic motivation in the studied group, based on assessment and environmental pressure, with a limited share of autonomic motivation. The results indicate the need to strengthen intrinsic motivation and psychological activities aimed at reducing emotional tension and improving self-acceptance.

## 4 Discussion

Our study of the motivation to lose weight among overweight and obese children and adolescents, conducted in three different environments (educational, clinical and spa), identified a number of important relationships. According to the results obtained, most participants made attempts to restrict food and follow dietary rules, but these actions were often short-term. Only 16% of the subjects maintained their diet for a full 28 days, which suggests a limited durability of motivation. Differences in emotional experience were also observed depending on the environment. High school students were more likely to report fear of losing control, feelings of guilt after eating and discomfort with their bodies than medical patients. Over 72% of participants felt uncomfortable with regular weighing and as many as 68% of respondents admitted that their body weight affects the way they perceive themselves. The results of our survey indicate that overweight and obese children have difficulty maintaining healthy eating habits – as many as 31% of respondents said they gave up trying after a few days. This may indicate a low level of internal motivation and a lack of support from the environment, as many of the children’s statements also revealed frustration, shame and a lack of belief in their own abilities, which may indicate a low level of internal motivation. In addition, some children indicated that they are unable to follow dietary recommendations without parental support, which emphasizes the role of family relationships in the treatment process. Although statistical analysis showed a significant relationship only between BMI and the level of dissatisfaction with weight (*p* = 0.0261), qualitative results indicate the possible role of emotional support from the family as a protective factor. During individual interviews, participants often spontaneously pointed out the influence of their parents’ attitude on their commitment and well-being, which may be a starting point for further research in this area. Therefore, it can be seen that emotional support from parents can play a key role in encouraging children and adolescents to adopt and maintain healthy eating habits. Children who feel accepted and understood by their loved ones can engage more openly in the process of lifestyle change.

According to current knowledge, therapeutic interventions that take into account the family context are much more effective than interventions based solely on the child’s individual involvement (28). Our own research, conducted in three different environments – educational, clinical and spa – clearly shows that the emotional and organizational involvement of parents and their attitudes towards health and lifestyle significantly increase the child’s motivation to change and promote the durability of therapeutic effects. These assumptions are consistent with previous literature reports, which indicate that children who receive active family support are more willing to undertake and continue health-promoting activities (29–31). However, the role of parents goes beyond mere control – they also act as role models, influencing the formation of behavior patterns and building a sense of agency and intrinsic motivation in the child. In addition, as shown by the qualitative data, children who reported greater emotional support were less likely to experience anxiety about losing control over eating and less likely to associate self-esteem with body image. These phenomena seem to be important not only from the perspective of the effectiveness of weight loss interventions, but also in the context of the prevention of eating disorders and emotional problems that often coexist with overweight in developmental age. Limiting the number of meals has a negative impact on the mental health of children and adolescents, leading to feelings of guilt (32). To prevent this, parents should set a good example by eating meals together with their children from an early age. This has a positive effect on the development of healthy eating habits (33–35). However, parental pressure – even unintentional – can create an environment in which the child begins to avoid eating, which in turn is associated with a higher risk of developing eating disorders (36).

In a study of Hispanic adolescents aged 10–14 years, they emphasized the importance of parents and children making decisions together as an important protective factor in the context of overweight and obesity. An analysis of data from two studies focusing on the risk of type II diabetes showed that adolescents who rarely or never participated in joint decision-making with their parents had more than three times the risk of obesity (OR = 3.50; 95% CI [1.25–9.83]) compared to their peers, who always made decisions together (37). In addition, many parents perceive childhood obesity as a sign of health, which is due to deeply rooted cultural norms, which can affect children’s inability to control their food intake (38, 39).

The data in the table ‘Respondents’ answers to the question ‘Does your weight affect the way you think of yourself?’ taking into account BMI’ are also noteworthy. The vast majority of overweight and obese children surveyed admit that their weight negatively affects the way they think of themselves. The way a child perceives their own body and their self-esteem are directly related to the atmosphere at home. A lack of support, excessive criticism and comparing the child to others can result in low self-esteem. Emotional support from parents can have a protective function – children who are accepted despite being overweight show greater emotional resilience, cope better with failure and are more committed to the healing process (40). The effectiveness of health measures is higher in children who receive adequate social support, and the family remains the main source of support during the developmental age. Sjunnestrand and co-authors emphasized the need to provide parents with adequate support, including issues related to conducting conversations about the child’s body weight and shaping constructive communication in this area (41). Although such conversations are mainly motivated by parents’ concern for their child’s health, they can be perceived by teenagers as critical, embarrassing or judgmental. As a result, they not only lead to lower self-esteem, but also increase the risk of developing behaviors such as restrictive diets, binge eating or avoiding meals. Research indicates that parents’ comments regarding body appearance and weight, especially during adolescence, can reinforce a negative body image and increase susceptibility to eating disorders in adulthood. For this reason, there is an increasing emphasis on the need to support parents in having empathetic, non-judgmental conversations with their children about health and lifestyle (42–46). It is worth noting that independent attempts to introduce dietary restrictions without proper support can lead to frustration, guilt and, consequently, unhealthy eating patterns, as noted by other authors (47–49). Therefore, it is important that interventions concerning obesity in children and adolescents, implemented from an early age, are directed at the whole family, which will help young adults to inhibit obsessive eating behaviors (50, 51). According to research by Skelton et al. active participation of parents in the process of controlling the child’s weight can contribute to better results in the reduction of overweight and obesity (52). Chan et al. also note that the role of parents’ presence during the weight loss process is an important factor in strengthening desirable eating habits in children (53). Interestingly, the study by Schmied et al. it was shown that low parental readiness to make health changes was a significant predictor of absence from intervention classes (OR = 0.41; *p* < 0.05), while a higher level of family functioning was associated with higher attendance (RR = 1.25; *p* < 0.01) (54).

Our results show that nutritional principles alone are not enough if the child does not feel supported. An effective intervention should combine emotional, educational and environmental support – not just restrictive dietary recommendations. How children eat and how they change their habits depends largely on the family environment, which indicates the importance of a family approach to obesity therapy (55, 56). The effectiveness of the family approach in the treatment of childhood obesity is confirmed by the results of the Fit Family Challenge study, in which primary care physicians implemented a family-centered behavior modification program. The intervention proved feasible and resulted in clinically significant changes in BMI and lifestyle of children, which emphasizes the role of the home environment and the availability of systemic support in obesity therapy (57). In addition, it is worth noting that in our study, children quickly became discouraged from adopting and maintaining healthy eating habits, which indicates the difficulty in maintaining motivation without adequate support from the environment. According to the results of previous studies, the inclusion of family-centered care principles in weight control interventions can not only increase the effectiveness and quality of treatment, but also reduce dropout rates from therapeutic programs (58). One of the positive effects of parents’ active involvement in the treatment of childhood obesity is the observed improvement in body mass index (BMI), which emphasizes the importance of a family approach in therapeutic interventions (59–61).

Our study has revealed many important observations, but it also has several significant limitations. Firstly, the use of self-reported questionnaires and participant declarations may introduce cognitive bias and a tendency to provide socially desirable answers. Secondly, the cross-sectional design prevents any firm conclusions about cause-and-effect relationships between the variables. Another limitation is the absence of a fully standardized tool to measure family support, even though the interviews clearly highlighted its relevance. Consequently, the conclusions concerning the influence of the home environment should be regarded as primarily observational and qualitative.

Furthermore, although the questionnaire was developed on the basis of established theoretical frameworks and validated instruments, most notably the Eating Disorder Examination Questionnaire (EDE-Q) originally validated in Belgium, this study represents only an initial step toward its cultural and linguistic adaptation to the Polish context. The full validation of the Polish version is still ongoing, and therefore the present findings regarding motivational and emotional factors should be interpreted with caution. Finally, the relatively small sample size and the recruitment from three specific environments limit the generalizability of the results to the broader population of overweight and obese children and adolescents.

Despite these limitations, the study provides valuable insights into the interplay between motivation, emotions and family support in the process of lifestyle change among young people, while also pointing to the need for further longitudinal and validation research that includes both the perspectives of children and their caregivers.

Taken together, our findings highlight the central role of both individual motivation and family context in the process of weight reduction among children and adolescents. The importance of parental involvement observed in our study is consistent with evidence showing that family-based interventions lead to significant improvements in BMI and lifestyle outcomes (62, 63). Likewise, the difficulties in maintaining long-term dietary changes reported by participants align with previous research demonstrating that intrinsic motivation is strongly associated with healthier weight trajectories and that interventions grounded in Self-Determination Theory (SDT) foster sustainable behavior change (64–66). These converging data emphasize that nutritional recommendations alone are not sufficient if they are not supported by an appropriate emotional and environmental context. At the same time, methodological aspects require careful consideration: while our analyses provided important insights, future research with larger samples should apply advanced approaches such as multilevel regression or structural equation modelling to capture the complexity of motivational and family-related factors influencing weight management. By combining individual-level motivation with structured family support and more rigorous analytical frameworks, future interventions may achieve stronger and more durable outcomes in the prevention and treatment of childhood obesity.

Another methodological limitation is the use of chi-square tests for some cross-tabulations with multiple categories, which in places resulted in small cell sizes. To minimize this risk, logistic regression models were applied in parallel, providing a more robust statistical basis for interpretation. Nevertheless, future studies with larger samples should employ advanced statistical approaches such as multilevel regression or structural equation modelling to capture the complexity of motivational and family-related factors influencing weight reduction.

In conclusion, the results obtained emphasize the importance of a comprehensive approach to the treatment of overweight and obesity in children and adolescents, in which not only nutritional interventions play a key role, but also emotional support and the active involvement of the family in the process of change.

From a practical perspective, the results suggest that family members should be actively engaged in weight reduction programs. Parental involvement could include setting a positive example in dietary behaviors, encouraging regular physical activity, and providing emotional support during lifestyle changes. Structured family-based interventions, such as joint participation in nutrition workshops, supervised exercise sessions, or family counselling, may increase the effectiveness of weight management strategies in children and adolescents. Healthcare professionals and educators should therefore consider approaches that integrate families as partners in the therapeutic process, rather than focusing exclusively on the individual child.

Given the cross-sectional nature of the project, the presented results should be interpreted as co-occurring relationships, not cause-and-effect relationships. The analysis does not allow to determine the temporality of phenomena or the direction of interaction between family support, types of motivation and eating behaviors, nor does it exclude the influence of unobserved confounding factors and the possibility of reverse causality. This means that higher family support, stronger controlled motivation or the presence of amotivation are related to the reported behaviors and emotions, but we cannot conclude that they cause them. To verify the directions of dependencies and mediating mechanisms, prospective studies are necessary, preferably longitudinal and interventional, with repeated measurements of support and motivation and objective indicators of health behavior. Complementing the analyses with a broader set of confusing variables and triangulation of data sources could further reduce the risk of bias and strengthen the relevance of the findings. These findings can inform the development of family-centered and school-based obesity interventions that combine nutritional education with motivational and emotional support strategies. Strengthening parental involvement and school-based peer programs could enhance intrinsic motivation and sustainability of healthy behaviors.

## 5 Limitations

This study has several limitations that should be considered. Firstly, the data were collected using self-reported questionnaires, which may introduce recall bias and a tendency to provide socially desirable responses. Secondly, the cross-sectional design does not allow for causal inferences regarding the observed relationships. Thirdly, although interviews revealed the important role of parental attitudes, no fully standardized instrument was applied to quantify family support, which limits comparability with other studies. Furthermore, while the questionnaire was developed on the basis of validated instruments, most notably the *Eating Disorder Examination Questionnaire (EDE-Q)* originally validated in Belgium, the present work should be regarded as an initial step toward its cultural and linguistic adaptation to the Polish context. A full validation process is still ongoing; therefore, the findings concerning motivational and emotional factors should be interpreted with caution. Finally, the relatively small sample size and recruitment from specific educational, clinical, and rehabilitation settings restrict the generalizability of the results to the broader population of overweight and obese children and adolescents.

## 6 Conclusion

The results of this study clearly demonstrate that family support, both emotional and organizational, plays a central role in the effectiveness of weight loss interventions among overweight and obese children and adolescents. Participants who experienced acceptance, understanding, and joint engagement from their parents were more motivated, had higher self-esteem, and showed greater persistence in sustaining lifestyle changes. Conversely, children lacking adequate family involvement more often reported difficulties in following dietary recommendations, feelings of helplessness, and negative emotions related to body weight. Importantly, not only the presence of family support but also its quality and consistency proved to be crucial.

From a practical perspective, these findings highlight the need for systemic approaches to childhood obesity, with interventions directed not only at the child but also at parents and the entire family through education and support programs. Such strategies may enhance the durability of therapeutic effects and improve quality of life in young patients.

It should be emphasized that the study included only basic sociodemographic information such as age, sex, height, weight, and place of residence, which limits the ability to fully interpret the influence of environmental factors on motivation and perceived family support. The lack of detailed data on socioeconomic status, parental education, and family structure prevented adjustment for these potential confounders. Therefore, the results should be considered descriptive and interpreted with caution. Future research should incorporate a broader range of sociodemographic variables to allow for more precise assessment of their role in shaping motivation and the effectiveness of family support in promoting healthy behavior change.

The study also underscores a significant methodological gap in current research: the lack of uniform, standardized tools that integrate psychological, social, and family components alongside somatic parameters. Although our questionnaire was grounded in validated instruments such as the EDE-Q, the Polish adaptation of this tool is still underway. Thus, the present findings should be regarded as preliminary. Future studies should address this gap by employing fully validated measures, larger and more diverse samples, and advanced statistical approaches (e.g., multilevel regression, structural equation modelling) to better capture the complex interplay between motivational, emotional, and family-related factors.

Ultimately, tackling childhood obesity requires an interdisciplinary effort—bringing together medicine, psychology, pedagogy, dietetics, and sociology—with the family positioned as a key partner in both prevention and therapy.

## Data Availability

The raw data supporting the conclusions of this article will be made available by the authors, without undue reservation.
